# A wide range of abiotic and biotic variables leaves most variation in bird nest architecture unexplained

**DOI:** 10.1098/rspb.2025.2013

**Published:** 2025-10-29

**Authors:** Michał T. Jezierski, Roger B. J. Benson, William J. Smith, Erin E. Saupe, Sonya M. Clegg

**Affiliations:** ^1^ School of Geography, Earth and Environmental Sciences, University of Birmingham, Birmingham B15 2TT, UK; ^2^ Division of Paleontology, American Museum of Natural History, New York 10024-5102, USA; ^3^ Department of Earth Sciences, University of Oxford, Oxford OX1 3AN, UK; ^4^ School of Life Sciences, University of Nottingham, Nottingham NG7 2RD, UK; ^5^ Department of Biology, University of Oxford, Oxford OX1 3PS, UK; ^6^ Helsinki Institute of Life Sciences, University of Helsinki, Helsinki 00790, Finland

**Keywords:** nest, birds, biotic interactions, environment, reproduction, life history, macroevolution, island, climate

## Abstract

Nests are the locations or containers for offspring, and mediate interactions between offspring and the environment. However, understanding how environmental factors shape the evolution of nest architecture is complicated. In particular, the relative contributions of biotic (e.g. protection from predation) and abiotic (e.g. microclimate maintenance) factors to the evolution of nest architecture have not been clearly quantified, and multiple nest traits, such as their location or shape, are rarely considered together. Here, we use a dataset of 3685 bird species for which data exist across multiple nest traits to characterize a multivariate ‘morphospace’ of nest architecture and use phylogenetic comparative methods to explore whether abiotic or biotic factors better explain this variation. We detect that abiotic environmental factors (climate) explain more variation than biotic factors. However, substantial variation in nest architecture remains unexplained after accounting for the variables used here, suggesting that commonly used nest traits may not capture covariation between nest architecture and the environment as expected. Nonetheless, our study demonstrates how nest evolution is affected by the environment on a global scale. This provides a foundation to explore a more diverse array of nest traits and environmental variables, to better understand nest evolution in the world’s birds.

## 1. Introduction

Nests are the locations or containers for the development of offspring [1] across a wide diversity of animals [2]. Nest architecture results from the heritable construction and site selection behaviour [1] and is among the most widely recognized examples of an extended phenotype—an external manifestation of an organism’s observable traits that directly affects its fitness [3]. The nest mediates the interaction of offspring with the environment and is the sole site for the earliest and most vulnerable stages of life [4]. All bird species use nests as sites of egg development, and most species, including all passerines, have nidicolous offspring that remain in the nest for an extended period after hatching. Important events of early avian development occur in the nest, with life-long fitness consequences [4]. Therefore, nest architecture should be under strong selection to optimize offspring survival and development [1,2,5].

Bird nests come in a variety of forms, from the elaborate arboreal hanging domes of the sociable weaver (*Philetairus socius*) to the placement of a single egg on a substrate by white terns (*Gygis alba*) [1,6]. Wide variation in nest form may even occur within a single family, such as ovenbirds (Furnariidae, Passeriformes), in which nests range from domes to cups to simple platforms [7]. The environmental correlates of evolutionary variation in nest form are now a major area of inquiry [5,8–10]. Numerous studies have explored the structural roles [9], thermal benefits [9,11,12] and protection from predators [13,14] provided by bird nests. Regional, multi-species comparisons have shown that certain aspects of nest architecture may correlate with climate [15,16], predator occurrence [12,17], or both, including variation in nest height [17,18], orientation [19] and site selection [13]. Most recently, global datasets [20,21] suggested that the evolution of univariate nest traits (e.g. structure or location, considered on their own) is shaped by climatic variables at a global scale [21]. Empirical work on the correlates of nest evolution has primarily focused on abiotic factors such as climate [21,22], with relatively little attention given to biotic factors or consideration of the relative importance of the two [4,23]. Previous work found that aspects of nest function, such as structural support in Australian birds [9] or thermal insulation in a global sample of a few hundred species [12], are supported as the primary functions of nests, over biotic factors such as protection from predators. These studies also identify body mass as a potentially important predictor of nest architecture. Body mass can affect nest architecture directly (e.g. lighter species can use finer materials in their nest construction), or indirectly (e.g. larger species are more likely to occur in high-latitude environments, where certain nesting strategies are not possible). Establishing the relative importance of potential covariates has remained difficult, since quantifying biotic interactions at a global scale is challenging compared with readily available data on abiotic conditions [24].

Analyses of nest architecture [21,25] typically focus on univariate nest traits, such as shape (e.g. cup or enclosed), site (e.g. vegetation or ground), attachment (e.g. basal or hanging) or height above ground (though see [16,26]). These traits are often considered independently and even coded as binary variables in statistical models (e.g. cup or not-cup) owing to the inherent difficulty of handling multivariate categorical data. However, nest traits might not evolve independently of each other because nest function is likely the outcome of multiple, interacting traits [25]. For example, nest shape coevolves with nest placement in old world babblers (Timaliidae), with enclosed nests correlating with ground placement and cup nests correlating with elevated nest placement [26].

Here, we consider multiple nest traits together using a newly collated dataset of bird nest architecture spanning all extant bird species (10 528 species according to Clements' taxonomy [27]). The dataset contains descriptions of five commonly used [20,21,25] nest traits: (i) nest type, describing whether the nest is in a cavity or not, (ii) nest structure, (iii) nest site, (iv) nest attachment, and (v) nest height. We use this dataset to represent nest architecture as a multivariate trait using ordination methods on 3685 species for which we have complete data for each nest trait and environmental covariables of interest. Our approach differs from previous global comparative studies of nest architecture in being the largest scale multivariate study of nest architecture to date, and in explicitly comparing the relative role of abiotic and biotic factors. We use multivariate phylogenetic analyses to investigate the relative impact of body size, abiotic (climate) and biotic factors on nest architecture, including vegetation availability, diversity of potential predators (as a proxy for predation risk) and island endemism (as a proxy for decreased competition and predation on islands). We also compare these results with univariate analyses on the same set of species, to show that multivariate approaches capture different axes of co-variation between nest architecture and the broader environment than most studies have reported to date.

## 2. Methods

### (a) Data collection

We collated data on nest architecture for 10 528 species of extant birds using *Birds of the World* [6]. We documented variation at the species level following previous functional trait studies [28,29]. We chose five traits based on previous studies of nest architecture [25,30], including nest type (cavity | not cavity); nest structure (scrape | platform | cup | enclosed); nest site (vegetation | cliff | ground | water body | underground | ant nest); nest attachment (basal | lateral | pensile) and the minimum and maximum recorded nest height (in metres (m)). Character states for each of these traits are described in table 1. All traits were coded by one author (M.T.J.) for consistency. We did not consider intraspecific variation in nest architecture, to avoid biases caused by differential research effort across species, species traits (e.g. missing data for species nesting high in cavities) and regions of the world. In cases where variation within species was indicated, we coded the most frequent trait. If no qualitative weight was given to frequency of the mentioned nest traits, the one mentioned first was selected. We also did not include brood parasites in our analyses owing to the diversity of nests they may use. We did not discriminate between natural and artificial nests, coding them with the most appropriate nest traits. A trait was considered missing if any aspect of the description was unclear or absent. When the distinction was made, we only coded nests used for breeding, and not for display or roosting. For each species, we recorded taxonomic information (order, family, genus and species), whether the breeding range was exclusively insular, and the minimum and maximum body size (in grams (g)) using measurements of adult sexed birds, where available. The database was collated between October 2020 and April 2021. *Birds of the World* [6] is constantly updated, and the nest traits were based on the versions of species accounts available between these dates.

**Table 1.** Character states of the four categorical nest traits (nest type, structure, site and attachment). Character states are based on descriptions in [25], with modifications based on recommendations in [30]. Nest height was additionally categorized for down-stream analyses, as described in the sections below.

nest type	non-cavity	nest not placed in any soil, rocky or wooden cavity
	cavity	nests located in soil, rocky or wooden cavities, whether excavated by the bird or not
nest structure	scrape	eggs were laid in a place with no obvious nest construction or with only brief scratching or cleaning
	platform	a nest where feathers, leaves, sticks, vines, dirt or other materials were stacked or loosely intertwined to form a platform
	cup	a nest with an erected, surrounding rim made by interweaving nest materials or mud; cup nests could vary in their depth, but parental birds could not hide their whole bodies inside the nests
	enclosed	nests where parental birds could sit inside without exposing any part of their bodies
nest site	ground	nest is located on the ground
	vegetation	nest located in/on trees, bushes, bamboo or thick tangled herbaceous vegetation (such as vines or reeds) that occupies forest understory, grassland, or wetland habitats
	cliff	nests placed in cliffs, river banks or piles of soil or rocks
	underground	nests built in burrows underground
	water bodies	nests built on the surface of water or piled up from the bottom of lakes or ponds and thus surrounded by water
	ant nest	nests built in termite or ant nests
nest attachment	basal	nests supported mainly from their bottom, including those sitting among multiple interweaving trees or bush branches and those inside cavities
	lateral	nests attached to supporting objects such as branches or rocks solely by their lateral parts
	pensile	nests hung down from a supporting object with its upper, narrower attachment part

The nest dataset was initially organized following the Clements/eBird 2019 [27] taxonomy used in the *Birds of the World* [6]. To maintain compatibility, mismatches with IUCN/BirdLife taxonomy [31] were identified and corrected using Avibase [32] (https://avibase.bsc-eoc.org/avibase.jsp) and original names provided alongside the data. We also identified each species as belonging to one of three broad avian groups: passerines, non-passerine landbirds and seabirds. Passerine birds were all members of Passeriformes. A bird was classified as a seabird if it consistently spends most time in marine habitats (coastal water, offshore waters, pelagic waters, but not the coastline, e.g. beaches) during a part of its annual cycle. All other birds not meeting these conditions were classified as non-passerine landbirds (see electronic supplementary material). Note there are no known marine passerines, therefore each category is exclusive.

### (b) Software used

All analyses, graphing and data preparation were done using R v.4.4.2 [33]. Data preparation relied on base operations and *tidyverse* v.2.0.0 [34]. Spatial data handling was done using packages *sf* v.1.0−19 [35] and *terra* v.1.8.54 [36]. Phylogenetic data were handled using *ape* v.5.8 [37] and *caper* v.1.0.3 [38]. Plotting was done using *ggplot2* v.3.5.1 [39], with additional use of *ggforce* v.0.5.4 [40] and *rphylopic* v.1.5.0 [41]. Specific analytical packages are referred to in appropriate sections. All original scripts and data are provided in the associated Dryad repository [42].

### (c) Describing nest architecture as a multivariate trait

To describe nest architecture as a multivariate trait, we used metric scaling in the form of principal coordinates analysis (PCoA). We only used species for which we had data across all five nest traits (i.e. type, structure, site, attachment and minimum and maximum height), as data imputation for categorical variables is associated with high uncertainties [43]. This resulted in a dataset of *n* = 3685 species, henceforth referred to as the morphospace dataset. The morphospace dataset is phylogenetically representative, containing nearly 40% of all species, 39 out of 41 bird orders, and 211 out of 246 bird families present in the Clements taxonomy of 2019 [27] (electronic supplementary material, S1). Within it, there are 2274 species of passerines and 1411 species of non-passerines (of which 127 are seabirds).

To ensure that nest height is on an equivalent axis of variation to the other traits, we recoded this continuous trait as a categorical trait by first calculating the geometric mean of nest height using the minimum and maximum height measurements for all species in the morphospace dataset. We considered ground-level height (0 m) as one category. For all non-ground-level nesting species (*n* = 2394), we calculated the 10th (1 m) and 90th (12 m) percentiles of nest height among species. We then classified the lowest 10th as ‘low’ nesting, the inner 80th as ‘medium’ nesting, and the highest 10th as ‘high’ nesting (for the distribution, see electronic supplementary material, figure S1). We recoded ant nests (*n* = 4 species in the morphospace dataset) as vegetation nests (all such nests remaining in this dataset were in ant nests in vegetation) and water body nests (*n* = 55) as ground nests, to remove rare (*n* < 100) nest traits for metric scaling analysis.

To perform PCoA, we calculated a Gower distance matrix using the package *cluster* v.2.1.3 [44] via the daisy() function with default settings. The distance matrix was then passed to the pcoa() function of the R package *ape*.

### (d) Multivariate phylogenetic regression

We tested abiotic and biotic correlates of nest architecture evolution using multivariate phylogenetic modelling. We used the principal components of climatic variables (described below) as abiotic predictors. Normalized difference vegetation index (NDVI), diversity of potential predators and island endemism were input as biotic predictors. We also considered the geometric mean body mass of the species, to account for the need for structural support provided by nests [9]. The geometric mean species’ body mass was calculated using the minimum and maximum values of body mass from *Birds of the World* [6].

In all phylogenetic models reported in this paper, we relied on the new phylogeny [45] from the Open Tree of Life framework [46], which is complete for most species of birds recognized in the Clements taxonomy [45]. We used the maximum clade credibility tree based on the Clements taxonomy version from 2021.

#### (i) Climatic principal components and vegetation availability

We characterized climate variables for all 3685 species in our morphospace dataset by taking annual averages of the following five monthly variables from WorldClim [24]: average temperature, precipitation, wind speed, water vapour pressure and solar radiation. Variables were averaged across a species’ breeding range, defined using BirdLife distribution maps v.2020 [47]. The breeding range included the total (i) year-round range and, where present separately, (ii) breeding range of the species (for details consult [47]) and was filtered to include only land areas using data from NaturalEarth (https://www.naturalearthdata.com/), following [48].

Climate data were obtained from WorldClim v.2.1 at 10-arcminute resolution [24]. The climate rasters were used to extract an average annual value for each climatic variable for each species. To reduce dimensionality, we used principal component analysis using the function prcomp() from the base *stats* R package [33]. The first two axes, which explained 84.74% of observed variation in climate (electronic supplementary material, figure S2), were retained for each species as a variable for phylogenetic modelling, following [16].

To account for the possibility that climatic variables are correlated with nest architecture owing to their impact on vegetation availability, we included NDVI as an explanatory variable. NDVI data were downloaded from NASA’s MODIS Terra mission [49,50] at https://neo.gsfc.nasa.gov/. Monthly resolution pictures for 2001 were downloaded to match the WorldClim data used, which is derived from 1970 to 2000 (NDVI is only available from 2001 onwards). Monthly NDVI estimates were averaged across the year, and then an average NDVI value from across each bird species’ breeding range was obtained.

#### (ii) Predator diversity

Data on actual predation pressure for birds do not exist at the scale required to examine macroevolutionary patterns across their entire radiation. Instead, we used the diversity of potential predators as a proxy for predation pressure, following [51]. This approach can lead to both underestimates (e.g. in cases where predator density is more important) or overestimates (e.g. in cases where predator species differ markedly in their rates of attack) of predation risk. However, previous comparative studies of birds have suggested the proxy can capture patterns at the broad spatial scales of interest here [51].

Potential predators were determined as all species of mammals, birds or reptiles known to include vertebrates in their diet (vertivores). To determine diet, we used EltonTraits [52] for mammals and birds and ReptTraits for reptiles [53]. We then matched each species to IUCN Red List range maps [31], resolving naming inconsistencies using the Red List and Avibase [32] for birds.

We calculated the average number of potential predators across a breeding bird’s geographic range at 10-arcminute resolution. To do so, we rasterized the ranges of potential predators derived from IUCN/Birdlife maps. We then summed the species richness across all predator groups for each grid cell and subsequently averaged species richness of potential predators across the breeding ranges of each bird species.

We tested our proxy for predation by examining the correlation between the morphospace dataset and a smaller dataset with more direct estimates of nest predation [54], using a phylogenetic generalized linear model. This analysis included all species present in the morphospace dataset that also had data on daily nest predation rates from [54] (*n* = 257 species). We used the package *phylolm* v.2.6.5 [55] to run this model, using a Brownian motion phylogenetic covariance structure. All explanatory variables were the same as for the multivariate phylogenetic model, including two climate principal component axes, NDVI, geometric mean body mass, predator diversity and island endemism. We simplified that model to retain only statistically significant terms. Body mass and predator diversity were statistically significantly correlated with daily nest predation, although the correlation was weak (total model marginal *R*^2^ = 0.04492, predator diversity marginal *R*^2^ = 0.02651) between our dataset and data on predation rates (electronic supplementary material, tables S1 and S2 and figure S3). While this correlation is weak, no other variable tested (other than body mass) is significantly correlated with a measure of nest predation. Therefore, we retain vertivore diversity as a metric of predation, albeit noting that its interpretation must be viewed in light of this weak correlation.

#### (iii) Island endemicity

Data on island endemicity were collected from *Birds of the World* [6]. The variable was binary (i.e. species were coded as island endemics or not). Species were considered island endemics if their entire breeding range was insular (islands being land masses up to and including Greenland). Our definition of breeding range allows for species that breed on islands, but winter elsewhere, to be considered as island endemics, since their nesting occurs in an insular environment—this includes seabirds and migratory species [48]. Island endemism was included because island species are considered to face distinctive biotic conditions, including lower inter- and higher intra-specific competition, and lower predation [56].

#### (iv) Model framework

For the 3685 species in our morphospace dataset, we investigated the impact of abiotic, biotic and body mass variables on nest architecture using multivariate phylogenetic regression models. This analysis was implemented using the *geomorph* v.4.0.9 [57] function procD.pgls(). Use of this method, as opposed to univariate approaches, allows us to model nest architecture as a multivariate trait. The model uses all non-zero axes of the PCoA as the response variable (Axis 1–Axis 12, electronic supplementary material, table S3) and therefore correlates the entirety of nest architecture (with interdependencies between traits) with the examined variables.

As we were interested in the relative contribution of each variable to explaining the variance in nest architecture, we did not use model simplification. The model formula used was:


nestarchitecture∼ climatePC1+climatePC2+predatordiversity+islandendemicity+NDVI+geometricmeanmass

The six predictor variables were tested for collinearity using variance inflation factors implemented in the R package *car* v.3.1.1 [58]. All variables had VIF 5.2 or lower, which is moderately stringent in avoiding false positives [59]. All variables were scaled and centred. The model was investigated for statistical issues by plotting the residuals, raising no concerns.

The multidimensional nature of the model makes it less straightforward to visualize how each predictor variable affects the discrete nest traits used to construct the nest architecture morphospace. To translate the multivariate model into interpretable relationships between traits and predictors we calculated regression scores for each species. Regression scores for a given predictor variable are calculated by projecting species' nest data orthogonally onto the axis in multivariate space, defined by the coefficients of that variable in the model. They provide a univariate index of where each species falls along the modelled relationship between a predictor variable and nest architecture. The further a regression score is from zero, the stronger the relationship between those nest morphospace values and the predictor variable. The direction represents the sign of the correlation. The regression scores can then be plotted, and individual nest traits highlighted, to show whether individual nest traits associate with predictor variables (e.g. by showing that all cavity nests are always on the positive side of the correlation between nest morphospace and a given variable, regardless of the other nest traits in a species). To give weight to these associations, we define an association as strong if all species with a given nest trait fall entirely on one side of a zero score (i.e. 0 indicates no correlation). We consider there to be a tendency for association when the interquartile range falls on one side of zero, but outlying values may cross zero.

Finally, we calculated the phylogenetic signal of nest architecture. We used the physignal() function of *geomorph* [57] to estimate the multivariate version of Blomberg’s *K* statistic across 1000 permutation iterations of the morphospace data at the tips of the phylogenetic tree [60]. Therefore, the reported *p*-value corresponds to the inverse of the number of permutation iterations. The function tests whether the phylogenetic signal for a given trait is statistically different from what would be expected of a phylogeny of the same size that evolved purely under Brownian motion [60].

#### e) Logistic model comparison

To explore congruence between our multivariate approaches and with univariate approaches used to discover correlates of nest traits in previous work (e.g. [21]), we used phylogenetic logistic regression following the approach of Sheard *et al*. [21]. To this end, we used the package *phylolm* v.2.6.5 [55], applying maximized penalized likelihood approach of the function phyloglm(). All nest traits (except cavity | non-cavity, which are naturally binomial) were recoded as present/absent to generate a binomial response variable. Every nest trait state (*n* = 17) was then tested individually using the generic formula:


nest trait present | absent ∼ climate PC1+climate PC2+predator diversity+island endemicity+NDVI+geometric mean mass

To ensure comparability with our multivariate analyses, we used the morphospace dataset for these models (*n* = 3684 species). The Common Ostrich (*Struthio camelus*) was removed from the dataset because it skewed residual distribution, and the resultant model coefficients, owing to its high mass (the same issue does not occur in procD.pgls analyses above owing to differences in the modelling framework).

## 3. Results

### (a) Multivariate organization of bird nest architecture

**Figure 1.**
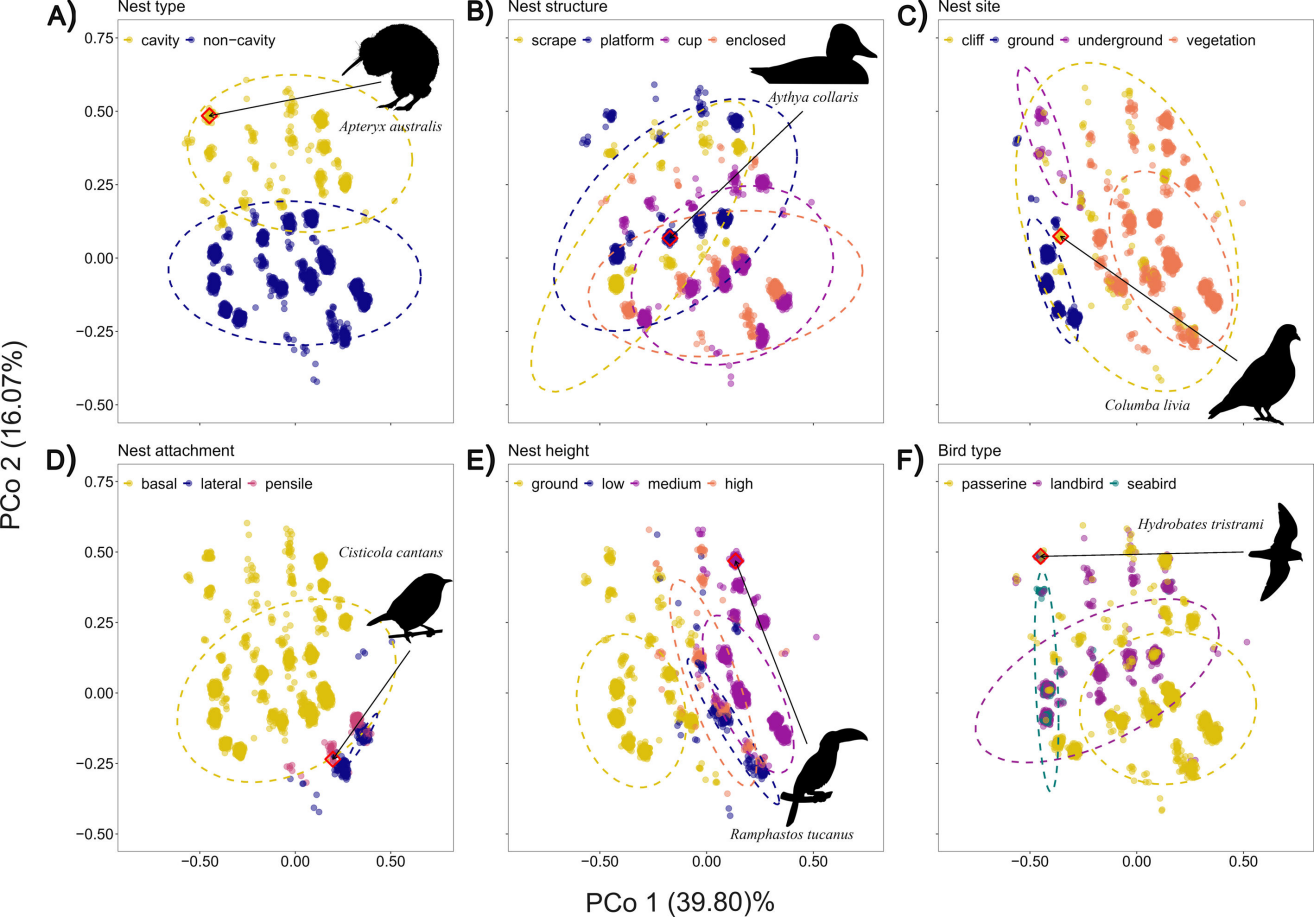
The diversity of nest architecture in birds. Nest architecture morphospace shown for the 3685 species of birds in the morphospace dataset. Owing to sharing of categorical nest traits, points can completely overlap among species, hence jitter was added for clarity using random normally distributed noise of s.d. = 0.01. Jitter was generated at plot-level, hence points may appear in slightly different positions between panels, but all panels present the same morphospace. Each subplot represents the same morphospace (i.e. all points/species are the same throughout) and is coloured according to trait. (A–E) show the distribution of each of the five nest traits and (F) shows the three broad exclusive groupings of birds (passerines, non-passerine landbirds and seabirds). Example bird species are featured in each plot using silhouettes. Each species' position in the morphospace is indicated with a line to a red diamond around their position. Silhouettes, from Phylopic [41], were committed to public domain by S. Traver, A. Wilson, F. Degrange, A. Vong, and L. Bobman.

The PCoA used to create the morphospace of nest architecture returned 12 axes of variation with non-zero eigenvalues (electronic supplementary material, table S3). The first four axes together explained 78.56% of all variation observed in nest architecture among the 3685 species in this dataset (see electronic supplementary material). PCoA axis 1 (PCo1, figure 1A–F) explained the most variation in nest architecture, at 39.80%. PCo1 primarily reflects the split between ground nests (negative scores) and elevated nests (positive scores), captured in nest site, height (figure 1C,E), and also nest attachment (as all hanging nests are elevated, figure 1D).

PCo2 explained 16.07% of the observed variation, and split nest architecture morphospace into cavity (most positive scores) and non-cavity (near-zero and negative scores) nesting birds (figure 1A). Moving along the diagonal of the two principal coordinate axes, hanging (lateral+pensile) nests are separated from basal nests, with only a few species having both cavity nests and lateral nests that separate from the main hanging nest cluster (e.g. bracket cups within a cavity of some swifts (*Chaetura*) and crag-martins (*Ptyonoprogne*)).

Variation in nest structure is not clearly organized by either PCo1 or PCo2, but is split by PCo3 and PCo4, with cup and platform nests forming a single grouping, and scrape and enclosed nests graphically split into two separate groups (electronic supplementary material, figure S4). The broad bird type groups (passerines, non-passerine landbirds and seabirds) did not have distinct nest architecture, although seabirds were primarily restricted to the part of the morphospace where ground-nesting species were found (figure 1F). Passerines reached the most extreme values of PCo1 and PCo2 owing to the occurrence of hanging nests in this group, placed both in cavities and in the open. Swifts (Apodiformes) also can have hanging nests in cavities, but passerines use more of the possible nest structures (figure 1D,F).

### (b) Evolutionary correlates of bird nest architecture

**Figure 2.**
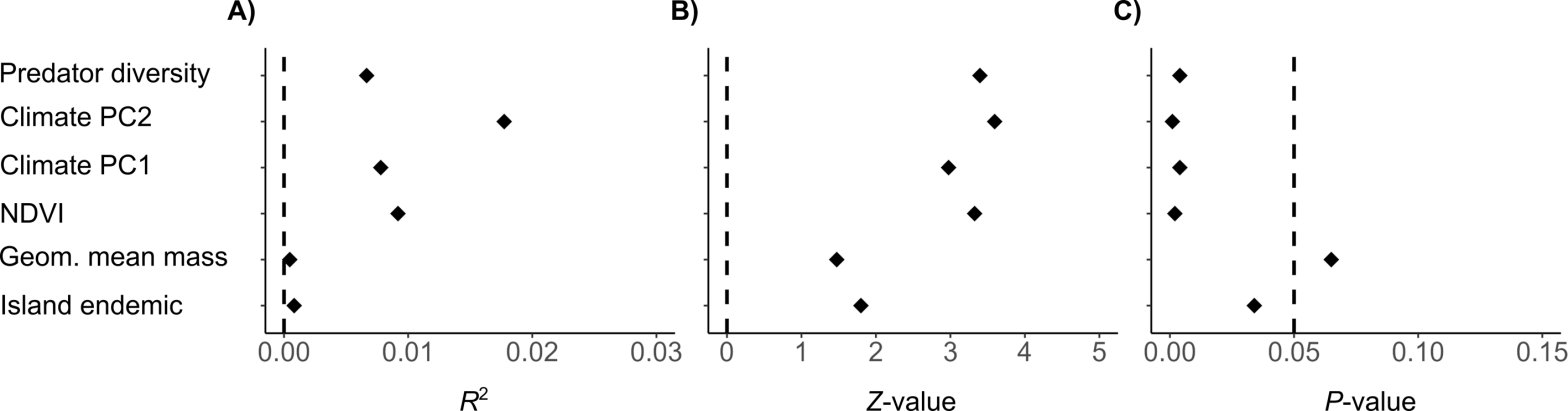
Multivariate phylogenetic linear model results. The model suggests that both abiotic and biotic factors correlate with nest architecture. (A), (B) and (C) correspond to the *R*^2^, *Z*-value effect size and *p*-values of each estimate, respectively, presented in electronic supplementary material in table S4. In (A) and (B), the vertical lines correspond to 0, and in (C), the line corresponds to 0.05, our significance threshold.

Modelling the correlates of evolutionary change across all 12 axes of nest morphospace shows that all predictor variables, except geometric mean body mass, are significantly correlated with nest architecture. However, the overall model only weakly explains the evolution of multivariate nest architecture (marginal *R*^2^ = 0.0427) (figure 2, electronic supplementary material, table S4). The two principal component axes of climate together explain approximately 2.5% of the variation in the model (figure 2A). These axes largely correspond to warm/wet to cold/dry conditions (PC1, marginal *R*^2^ = 0.008), and dry/windy to cold/wet/still conditions (PC2, marginal *R*^2^ = 0.018), with the latter being the single best predictor of nest architecture evolution. NDVI additionally explains 0.9% of the observed variation, which is more than climate PC1 (0.8%) alone. Total predator diversity explains 0.7% of the variation observed in nest architecture, island endemicity explains 0.08% and body mass explains 0.05% (electronic supplementary material, table S4).

**Figure 3.**
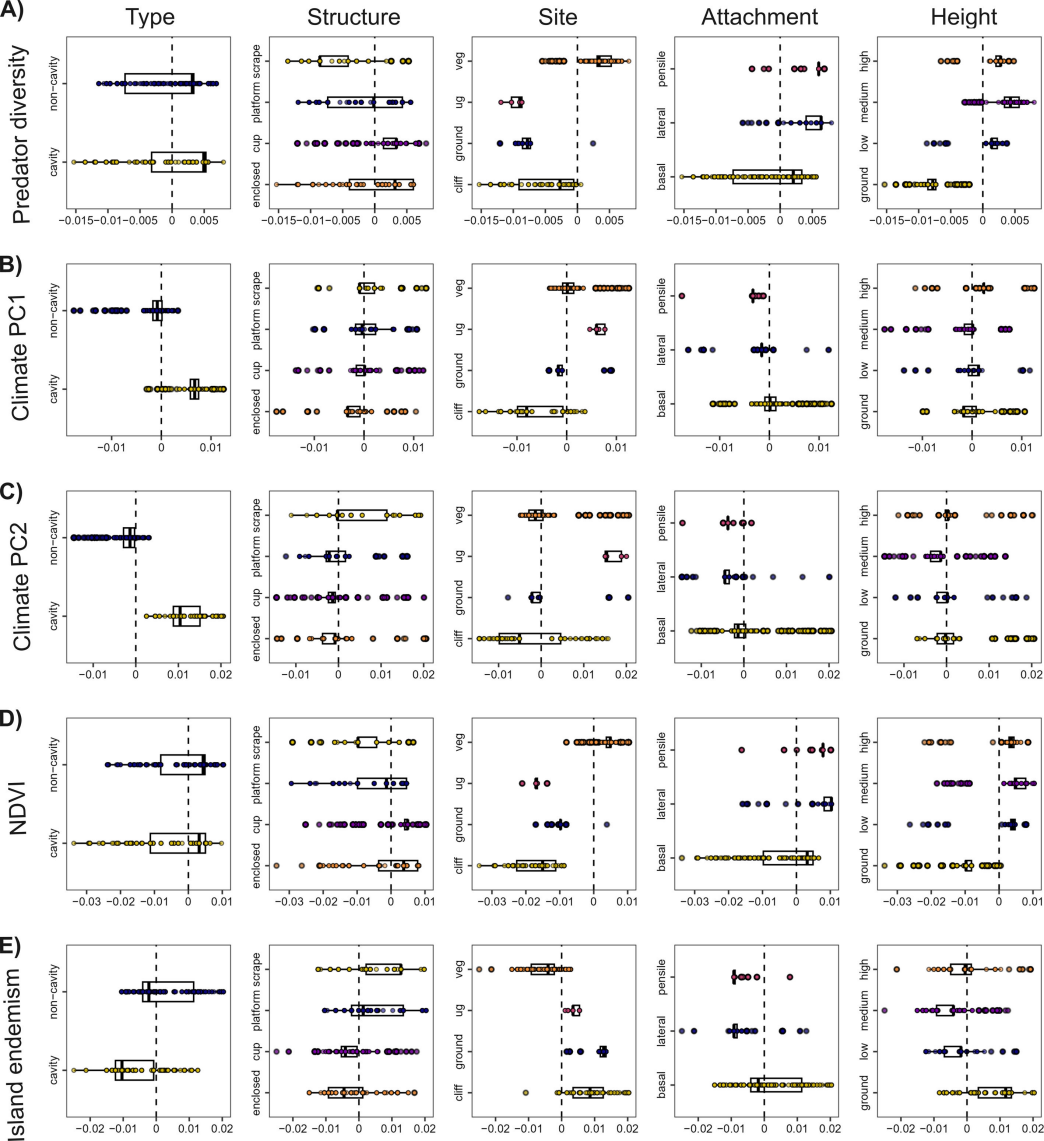
Associations of abiotic and biotic factors with individual nest traits from the multivariate phylogenetic model of nest architecture, shown using regression scores. Each plot represents the product of matrix multiplication of the 12 axes of nest architecture and the transposed coefficient for each predictor variable determined using a procD.pgls model (see §2d(iv)). The score for each variable (on the *x* axes) thus represents an association between the multivariate nest architecture and the predictor variable. The further away the value is from zero, the stronger the association, with the sign determining the direction of association (i.e. positive or negative). To illustrate univariate trait contributions, each boxplot presents the occurrence of a given nest trait, along the distribution of all multivariate nests for that predictor variable. We present results by predictor variable and nest trait for all significant terms of the model reported in figure 2. Each point represents a species, and the boxes represent the inter-quartile range across all species. Abbreviations used for nest site: ug: underground; veg: vegetation.

Although the overall proportion of variation explained by the model is low, we detected clear and significant correlations between nest architecture and both abiotic and biotic variables (figure 2). Using regression scores for each species (see §2), we visualized how individual components of the multivariate nest architecture (i.e. nest traits) associate with the examined environmental variables (figure 3).

Climate PC1 and PC2 (figure 3B,C) explained the most variation in the model. Higher values of PC1 (colder, drier, windier and less insolated) and PC2 (windier, hotter and more insolated) were both strongly positively associated with underground nests. Additionally, high values of PC2 were strongly positively associated with cavity nests, while high values of PC1 were strongly negatively associated with pensile nests. Across both climate axes, shared negative tendencies were observed for non-cavity, cup, enclosed, ground and lateral nests. Climate PC1 also tended to be negatively associated with cliff nests, and PC2 with medium-height and pensile nests.

Among biotic variables, NDVI was the strongest predictor in the model, with strong negative associations with underground and cliff nests (figure 3D). NDVI tended to associate positively with cup, vegetation, lateral, pensile and all elevated nest heights (low, medium, high) and negatively with scrape, ground and ground-height nests. Predator diversity (figure 3A) was strongly negatively associated with underground and ground-height nests. It showed positive tendencies for cup, lateral, hanging and elevated nests, and negative tendencies for scrape, ground and cliff nests. Finally, island endemism was strongly positively associated with ground and underground nests (figure 3E). Island endemism also tended to be positively associated with ground-height and cliff nests, and negatively associated with cavity, cup, vegetation, lateral, pensile, low and medium nests.

### (c) Phylogenetic signal in nest architecture

Nest architecture shows weaker phylogenetic signal than expected from a phylogeny of this size if evolving purely under Brownian motion processes (multivariate Blomberg’s *K* = 0.0852, *p*‐value = 0.001).

### (d) Uni- and multivariate modelling of nest architecture do not produce congruent results

The results of univariate analyses of nest traits are presented in electronic supplementary material, tables S5–S20. Despite using the same dataset, the results differ from those run with a multivariate framework. Logistic regressions returned a significant correlation (both positive and negative) of body mass with 11 out of 17 nest trait states. However, body mass is non-significant at *α* = 0.05 in our multivariate analysis. Of the associations and tendencies identified in the multivariate analyses (figures 2 and 3), only seven were also significant in univariate analyses and all related to correlations of nest traits with NDVI and island endemicity (e.g. correlation with vegetation nests; electronic supplementary material, tables S8, S11, S13, S15, S19, S20). Two associations with climate axes (PC1 and PC2) showed opposite signs in the univariate models, with positive correlations detected for cup and ground nests. Additionally, three traits (scrape, underground and pensile nests) showed no significant correlations in univariate analyses, despite showing clear associations in multivariate analysis. Basal nests were not associated with any predictor variable in the multivariate analyses, but were strongly correlated with body mass, both climate PCs and island endemicity in the univariate logistic analyses (electronic supplementary material, table S14).

## 4. Discussion

Despite accounting for a wide range of climatic and biotic factors, many of which had been hypothesized to affect nest architecture in birds [1,2,5], our models explained only a small fraction of the variation observed in multivariate nest architecture (marginal *R*^2^ = 0.0427). This limited explanatory power was observed even though we represented nest architecture as a multivariate trait—an approach that more realistically reflects the integrated nature of nests, in which multiple nest traits are expected to respond jointly to abiotic and biotic pressures [16,26]. We applied a multivariate framework to reveal previously unidentified axes of co-variation between nest traits and the abiotic and biotic environment, which were not observed in univariate analyses. We found that univariate models applied to the same dataset produced different conclusions regarding the association of biotic and abiotic variables with nest architecture.

### (a) Abiotic impacts on nest architecture

In our multivariate models, the two climatic predictors explained the most variation in nest architecture (marginal *R*^2^ = 0.008 for PC1; marginal *R*^2^ = 0.018 for PC2). This suggests that maintaining an appropriate microclimate may be a stronger selective pressure on nest traits on a global scale, and across numerous aspects of nest architecture, than biotic factors.

Our results indicate that cavity nests (including all underground nests) are strongly positively associated with harsher environments, characterized by extreme temperatures, aridity and wind. Previous studies have shown that enclosed nests (a character state for structure, describing a nest where a parental bird can sit without exposing its body) provide important thermal benefits [1,12,15]. Cavity nests could perform a similar role to enclosed nests, as they would typically also cover the adult individual. Cavity and underground nests gain insulation from both the nest materials and the surrounding cavity walls, likely resulting in robust protection from harsher environments associated with positive values of climate PCs. Such protection may include avoiding heat loss [13,61] and over-heating [62], as well as buffering against inclement weather. However, our analysis found that enclosed nests themselves tend to have a negative association with both climate axes, suggesting these are found in warmer and wetter environments instead. Therefore, we detect the previously shown relationships between climate and nest architecture, but for a different, although similar, element of nest architecture.

We observed a strong negative association of pensile nests, and tendencies for a negative association of non-cavity, cup, enclosed, ground, cliff and lateral nests, with both climate axes (PC1 and PC2). Many of these nest traits (e.g. cup, ground or cliff nests) may increase direct exposure to climate elements, which may be particularly detrimental in the windy and arid conditions denoted by positive values for both climate PCs. Hanging nests (lateral and pensile) are most often built in vegetation and are positively associated with vegetation availability in our models (see below; figure 3). However, hanging nests may also confer benefits in protection from predators, as supported in our analysis, and from brood parasites [63], so the association we recover here requires further investigation.

### (b) Biotic impacts on nest architecture

The biotic factors tested (vegetation availability, predator diversity and island endemicity) together explained less of the observed variation in multivariate nest architecture (marginal *R*^2^ combined = 0.0166) than the climate PCs together, and even less than climate PC2 alone. Vegetation availability was the strongest biotic predictor, followed by predator diversity, which we have shown to be weakly, but significantly positively, correlated with nest predation rate (figure 2). Island endemicity had the weakest explanatory power of all predictor variables, though it was still clearly associated with distinct patterns of trait distribution in the wider morphospace (figure 3).

NDVI is a widely used proxy for vegetation availability, which has been predicted to be an important driver of nest architecture [1]. However, macroecological studies have not tested this relationship directly, instead focusing on climatic variables (e.g. [15,16,21]), with the recognition that vegetation covaries with climate (e.g. sparse vegetation occurs often in hot-dry areas, which may force nest adaptation [15]). In our analysis, vegetation availability, as captured by NDVI, showed strong negative associations with cliff and underground nests, and tendencies for negative associations with scrape, ground and ground-height nests. These associations make intuitive sense, as lack of vegetation would prevent nesting in vegetation, as well as possibly limiting material for nest building, resulting in scrape nests. This is further confirmed by tendencies for positive associations for cup, vegetation, hanging and elevated (low, medium and high) nests, suggesting that vegetation availability may be an important driver across multiple elements of nest architecture. Positive associations are particularly strong with many nest architecture elements characterizing passerine birds (e.g. cup and hanging nests).

The statistical associations between nest architecture and predator diversity suggested a role for nest architecture in preventing access to predators. Nest traits that could potentially be more exposed to predators, such as cliff and underground nests, were strongly negatively associated with predator diversity, while ground, ground-height and scrape nests also tended to have negative associations. All of these nest traits may result in greater accessibility and visibility to predators, and indeed ground and ground-height nests are often expected to be at greater risk of predation [1,26] despite behavioural adjustments such as crypsis that may help to protect them [14]. Therefore, it follows that these nest traits would be more frequently used when predator threat is lower. Further supporting this assertion, we found tendencies for positive association with predator diversity of traits that may enhance concealment and place nests beyond the reach, or sight of, predators, such as cup, hanging and elevated nests. Despite the low explanatory power, these statistical associations align well with the expected role of nests in protection from predators. Indeed, increasing nest elevation has been implicated in one of the few documented cases of rapid evolutionary change in nest architecture. An island endemic, the O’ahu ‘Elepaio (*Chasiempis ibidis*), has increased their nesting height since the introduction of ground predators to Hawaii [17]. While our metric of predation pressure was only weakly correlated with daily nest predations, our results may suggest an important role for predation in shaping nest architecture. Future studies using more precise or comprehensive measures of predation risk could provide additional clarity on the strength of this biotic factor on nest architecture.

Island endemics face different biotic conditions than mainland species, including lower predation and interspecific competition [51,56]. In our analyses, island endemics showed strong positive associations with ground and underground nesting, and tendencies for positive associations with ground-height and cliff nests. Therefore, the statistical associations with island endemicity are opposite to those we find for predator diversity, and thus consistent with the hypothesis that island-related adaptations are, at least in part, driven by lower predation pressuree. Furthermore, island endemics showed tendencies for negative associations with cavity, cup, vegetation, hanging and elevated nests (except for high nests). These associations suggest a possible island syndrome, or a pattern of convergent evolution on islands [48,51,56], which seems to result in ‘simpler’ nests, at least in terms of their placement and modes of attachment. Cavity nests could also be considered a ‘simpler’ trait, providing more protection without the need to build additional structures (although some species may still build complex nests within cavities). However, we found a tendency for them to be negatively associated with island endemicity. Our analyses show that cavity nests are positively associated with harsher climates, and islands tend to have lower seasonal variation than continental areas at the same latitude [64]. This might imply a cost to cavity nesting, such that it is less advantageous under island conditions (of more stable climates and lower predation). Alternatively, island communities—and resultant patterns of trait occurrence on islands—can be strongly affected by filtering (i.e. traits are biased owing to likelihood of colonisation, not *in situ* evolution), and it is therefore possible that cavity nests, or any other negatively associated nest traits, are more prevalent in species with lower chances to colonize islands.

Finally, we did not observe a significant correlation between body mass and multivariate nest architecture. This was surprising because many of our univariate trait state models (11 out of 17) did show an association with body mass, and structural support, as measured by scaling of nest structural traits with body mass, has been shown to be the most likely driver of nest structure in a sample of cup-nesting Australian birds [9]. However, this apparent inconsistency could be owing to the nature of the data. Whilst specific nest traits (e.g. platforms) may be correlated with body mass when expressed binomially, multivariate analyses treat these traits as components of a broader architecture. For example, platform nests can be grouped with other nest structures that otherwise are all non-cavity, vegetation, basal, elevated nests. Because such multivariate combinations occur across the full spectrum of avian body mass, the signal of body mass itself is reduced, and perhaps indeed less important to multivariate nest architecture than climatic or other biotic variables. Additionally, owing to our categorical grouping, many axes of variation in structural traits relevant to body size may be ‘lumped’ in our analyses. For example, both brown gerygones (*Gerygone mouki*) and hamerkops (*Scopus umbretta*) build enclosed nests in vegetation according to our trait coding [6]. However, the two birds differ in body mass by two orders of magnitude [6], and the thickness, size and surface area of their nests, which were the actual traits shown to provide structural support [9], vary greatly between the two species. Including additional variables that describe wall structure, as was the focus in [9], may be more directly relevant to associations with body size. Finally, considering body mass more in-depth, e.g. by focusing on the mass of the incubating sex alone, could clarify the observed patterns.

### (c) Low phylogenetic signal and model explanatory power in nest architecture

We observed low phylogenetic signal (Blomberg’s *K* = 0.0852) compared with a null expectation of Brownian motion for a phylogeny of this size [60], meaning that closely related species are less similar in nest architecture than more distant relatives (or, that distant relatives are more similar than expected from the differences among close relatives). Low Blomberg’s *K* is interpreted as deviation from the expectation that closely related species should be more similar to one another in trait space, namely that they are over-dispersed [60,65,66]. This could be caused by the occurrence of frequent, large changes to nest traits among close relatives, as is the pattern observed in some groups, e.g. in nest structure in ovenbirds (Furnariidae) [7]. Greater-than-expected similarity of nest traits between distantly related groups could also be brought about by the existence of a limited trait space of nest architecture, such that distantly related species often explore similar regions of nest morphospace. Indeed, in our nest architecture morphospace, very distinct orders and functional groups share the trait space, suggesting high propensity to ‘repeat’ nest traits across distantly related groups. However, our test is currently limited only to evolution under Brownian motion, owing to methodological limitations in analyzing the phylogenetic signal in multivariate traits.

We found only weak effects of abiotic and biotic factors on nest architecture. This contrasts with studies for individual species or populations, which show that environmental factors may have very clear correlations with nest traits [12,13,18,19]. While macroevolutionary studies, such as ours, can identify clear trends in relation to broad-scale climatic or geographic variables [21,22], our study suggests that nest architecture evolution may be shaped by other processes not considered here. We propose several possible explanations for this. First, the environmental variables used here may not capture the microenvironmental pressures on nest traits. For example, there may be a direct trade-off between exposure to elements and protection from predators with relation to nest location [13], but such exposure varies with microenvironment (e.g. density of branches) rather than broad climatic variables. Second, it might be that the functional relationship of nest traits with the environment is not straightforward, resulting in multiple strategies being suitable in broadly similar conditions, the differences of which are determined by smaller-scale processes. For example, this could explain the coexistence of many different nesting strategies within a single ecological community. Hence, many macroevolutionary studies, including ours, may focus on environmental correlates that do not directly correspond to the environmental correlates affecting nest architecture. Third, large global analyses, such as this one, may also fail to consider variation in response across phylogenetic groups.

Notably, many previously reported correlations between abiotic and biotic factors, both at regional and global scales [12,13,18,19,21,22], have been found using univariate approaches. Multivariate methods, as used here, may capture different axes of variation between nests and the environment because nest traits covary [16,25], and changes in one nest trait may facilitate or constrain other traits [25,26]. We used phylogenetic logistic regressions, as in previous comprehensive studies of macroecological patterns in nest architecture [21], to explore the discrepancies between the two approaches. We found incongruent patterns between the multi- and univariate approaches, as discussed above with respect to body mass. These discrepancies suggest that multivariate approaches, as used here and in some prior studies [16,26], may offer a fundamentally different perspective on nest evolution compared with univariate approaches.

Finally, it is worth considering that the commonly used nest traits do not ideally capture the functional properties of nesting with the environment. Indeed, datasets with more detailed coding of nest traits (e.g. where our ‘enclosed’ category would be separated into categories such as ‘simple dome’ or ‘dome with tunnel’ or ‘pouch’) show clearer correlations in conjunction with environmental variables [10,16,63]. Many nest traits not considered here may have greater adaptive importance, such as the type of insulating material [11,67], size and proportions of nests [9,68], nest location within habitat (as opposed to just nest site) [13] and nest orientation [19]. Expanding our knowledge of these additional traits across the avian tree may provide more nuanced understanding of the drivers of nest architecture.

## 5. Conclusions

We provide the first global investigation of multivariate nest architecture, mirroring recent advances in our understanding of broad-scale patterns in avian morphological evolution [20,21]. We find that both abiotic and biotic variables clearly impact the evolution of nest architecture, despite overall weak correlations. We also find that multivariate nest architecture is characterized by weak phylogenetic signal under Brownian motion, implying that nest architecture is overdispersed. Our multivariate analyses lead to different conclusions about nest architecture evolution, from those of univariate analyses applied to the same dataset, indicating that our more holistic approach to nest architecture may provide a novel way to investigate nest evolution. As data incompleteness is resolved through continued study of avian natural history, and as other aspects of bird nest architecture are characterised (including nest size, materials orientation, microhabitat or concealment), we expect our database and approaches to provide a solid foundation to examine micro- and macroecological patterns in the evolution of avian nests.

## Data Availability

All original data and scripts, as well as a README detailing the use and access of publicly available datasets in the study, are to be found in a Dryad repository [42] Supplementary material is available online [69].
